# BART: bioinformatics array research tool

**DOI:** 10.1186/s12859-018-2308-x

**Published:** 2018-08-08

**Authors:** Maria Luisa Amaral, Galina A. Erikson, Maxim N. Shokhirev

**Affiliations:** The Razavi Newman Integrative Genomics and Bioinformatics Core, Salk Institute for Biological Studies, 10010 N Torrey Pines Rd, La Jolla, CA 92037 USA

**Keywords:** Microarray analysis, Differential expression, Online tool, R, Functional enrichment analysis, Automated analysis, Graphical user Interface, Gene expression omnibus

## Abstract

**Background:**

Microarray experiments comprise more than half of all series in the Gene Expression Omnibus (GEO). However, downloading and analyzing raw or semi-processed microarray data from GEO is not intuitive and requires manual error-prone analysis and a bioinformatics background. This is due to a lack of standardization in array platform fabrication as well as the lack of a simple interactive tool for clustering, plotting, differential expression testing, and testing for functional enrichment.

**Results:**

We introduce the Bioinformatics Array Research Tool (BART), an R Shiny web application that automates the microarray download and analysis process across diverse microarray platforms. It provides an intuitive interface, automatically downloads and parses data from GEO, suggests groupings of samples for differential expression testing, performs batch effect correction, outputs quality control plots, converts probe IDs, generates full lists of differentially expressed genes, and performs functional enrichment analysis. We show that BART enables a more comprehensive analysis of a wider range of microarray datasets on GEO by comparing it to four leading online microarray analysis tools.

**Conclusions:**

BART allows a scientist with no bioinformatics background to extract knowledge from their own microarray data or microarray experiments available from GEO. BART is functional on more microarray experiments and provides more comprehensive analyses than extant microarray analysis tools. BART is hosted on bart.salk.edu, includes a user tutorial, and is available for download from https://bitbucket.org/Luisa_amaral/bart.

## Background

A microarray is a powerful and cost-effective tool used to detect the gene expression of thousands of genes simultaneously. Biologists and clinicians use microarrays to determine gene expression levels under different conditions by measuring the binding of mRNA to oligonucleotides probes attached to chips. The Gene Expression Omnibus (GEO) is an online public repository for high-throughput expression data sets containing over 2 million samples grouped into over 90,000 individual series. GEO is maintained by the National Center for Biotechnology Information (NCBI) and has data organized into 4 components: platforms (GPL), samples (GSM), series (GSE), and DataSets (GDS) [5]. Series records (GSE) organize the samples into a meaningful experiment which can be analyzed to find differentially expressed genes between one or more conditions. GEO includes several basic analysis tools such as GDS tools and GEO2R. GDS tools provide clustering and differential expression testing for curated data on GEO, however, GDS is only available for some experiments with curated user-submitted expression tables. Although GDS can compare gene expression and perform clustering for some data sets, it does not perform generalized linear model differential expression testing, and is not available for most GEO series (currently less than 5% of all submitted series have GDS records). Similarly, GEO2R is a tool that performs differential expression testing on most platforms of GSEs [1]. GEO2R is limited to a single pairwise-comparison for differential expression testing, is only functional on certain datasets which have user-submitted expression tables of a limited size, and does not provide quality control plots or clustered heatmaps. A recently developed tool designed for microarray analysis, GEO2Enrichr, is a browser for extracting information from published microarray data on GEO, but it does not work on many microarray platforms, finds at most 1000 differential genes, and does not generate differential expression tables with *p*-values [7]. ShinyGEO is another microarray analysis tool which is functional on many microarray datasets but does not provide quality control plots and only performs differential expression testing for one gene at a time [4]. Since microarray datasets comprise the majority of expression datasets in GEO, since microarray assays are still popular for specific applications, and since extant tools for microarray analysis are limited, there is still a need for a free, flexible, easy-to-use microarray analysis toolkit for analyzing novel or reanalyzing published array data. Here we introduce BART: a freely available web tool that enables scientists and clinicians without bioinformatics knowledge to perform their own state-of-the-art customized analyses on a variety of microarray experiments using an intuitive interactive interface. We then show that BART provides a more informed and accurate analysis compared to four other popular microarray analysis tools using previously published human disease studies.

## Implementation

### Workflow overview

**Fig. 1:**
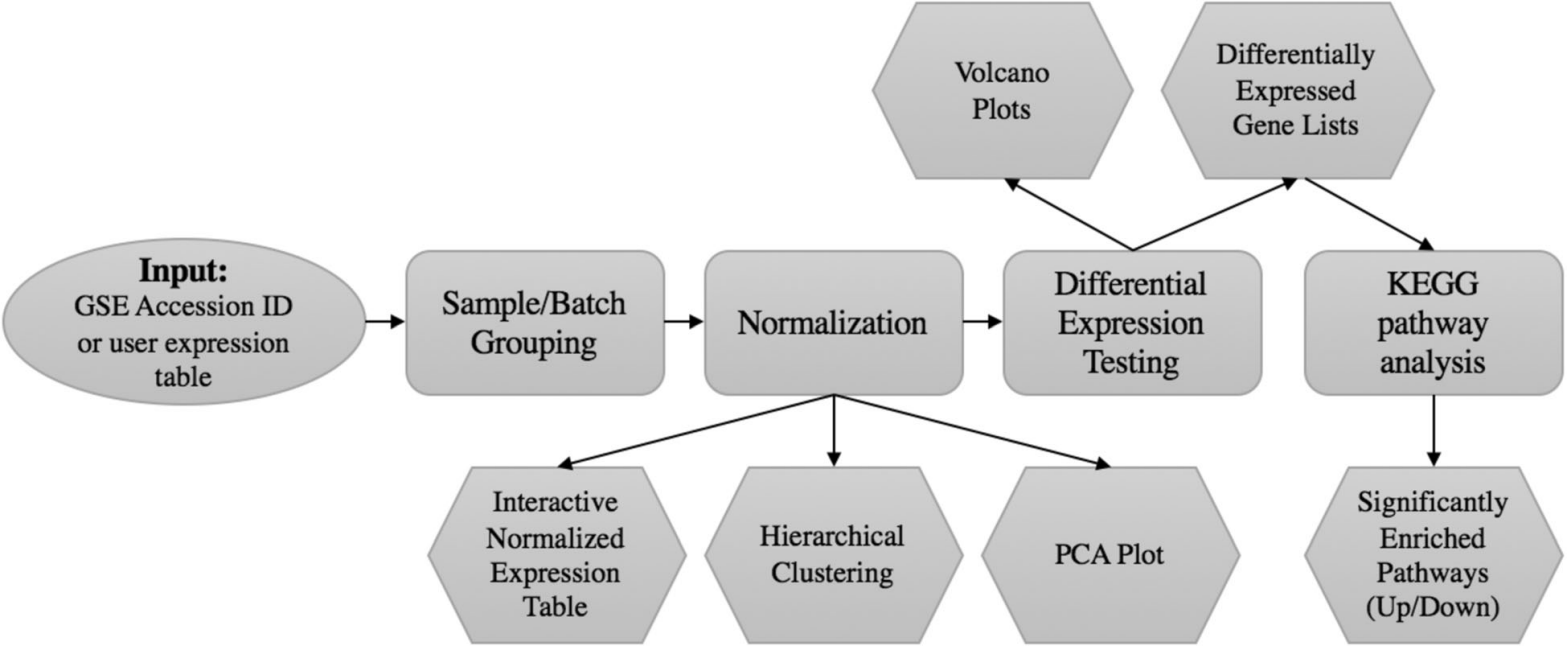
BART workflow. Multiple analyses can be carried out on a variety of input formats to identify sets of genes and associated biological processes that change between conditions

BART contains six modules that enable users to process raw microarray data from GEO or locally into a list of differential genes and associated pathways, enabling everyone to interpret microarray data in terms of underlying biological processes. Figure 1 summarizes the workflow from data import from CEL, GEO accession, or data matrix, through grouping by variable or feature, batch effect correction, normalization, visualization with heatmaps/PCA, differential expression testing, and finally functional enrichment. Users can access BART from our dedicated server (bart.salk.edu), or by downloading the associated R [12] Shiny code and running it locally.

### Data input

As input, BART requests a GSE accession ID which is typically found within publications, or by searching GEO online. BART automatically downloads the data from GEO and parses it for display in the user interface using the GEOquery R package [3]. Alternatively, a user can upload their own microarray expression table to BART for analysis. After loading data, BART suggests groupings for differential expression testing based on the sample characteristics described, such as treatment, genotype, or tissue. The user then selects which variable(s) should be used for grouping and indicates whether a batch effect correction is necessary. A batch effect correction may be important when comparing datasets with paired variables that include technical variability, such as different sample preparation protocols as demonstrated below. BART also accepts manual entry for grouping of samples and for batch effect correction in the editable metadata table that is displayed. If starting from a user expression table, BART accepts gzipped or uncompressed tables that contain unique identifiers in the first column, followed by expression values.

### Preprocessing

If raw fluorescence CEL files are used as input, BART detects whether to use the Affy [6] or Oligo [2] platforms and performs RMA normalization that attempts to remove local biases across samples in order to enable meaningful differential expression testing [8]. If a user expression table is uploaded, BART automatically detects whether a log2 transformation is needed and normalizes the data, which is important for reducing technical bias in downstream differential expression testing. The normalized expression table is then displayed in a searchable table and is available for download. A bar graph of the expression of each gene across conditions is generated to facilitate quick comparison of specific genes.

### Clustering and QC

Hierarchical clustering is a useful way to determine how well the samples/replicates in the experiment group together. The hclust R function is used to perform clustering of the top 1000 expressed normalized genes. In addition, Principal Component Analysis is used on the top 1000 expressed genes to transform and visualize samples as a function of the top two independent descriptive variables, which depict the most sample variance. Data points are labeled according to the desired grouping and can be scrolled over for sample information.

### Batch effect correction

Batch effects refer to any unwanted technical variation between samples that may have systematic effects on data. In microarrays, these effects are especially important to address because different gene chips and RNA isolation methods can produce unwanted technical variation between samples that could affect differential expression testing. These effects should be accounted for in microarray analysis so that the true biological differences between samples can be found. BART applies a batch effect correction in the design for differential expression testing if requested by the user by specifying a batch effect variable during differential expression testing.

### Differential expression and post-processing

BART leverages the LIMMA bioinformatics package [14] to perform differential expression testing. Results are annotated with gene names and gene symbols, as well as fold-changes and *p*-values adjusted for multiple testing for the full gene lists for each pairwise comparison of the groups specified. A link to the www.genecards.org entry [13] for each differentially expressed gene is also included in the differential expression table, allowing users to quickly learn more about specific genes of interest. In addition, volcano plots are generated for visualizing the differential expression data in terms of log2 fold-changes and adjusted *p*-values. BART automatically generates volcano plots for each differential expression comparison and highlights genes that are differentially expressed with adjusted p-values less than 0.05, which provides a quick global view of differential expression between conditions. Gene symbols, platform IDs, and coordinates are automatically shown when scrolling over each data point. All data and plots can then be downloaded for additional analysis and validation.

### Pathway enrichment analysis

The WebGestalt [16] R package is leveraged to perform over-representation analysis (ORA) of KEGG pathways within the significantly differentially expressed gene list produced by BART. A table of overrepresented KEGG pathways and bar graphs summarizing the list of significantly differentially expressed genes based on the Gene Ontology (GO) Slim datasets are displayed. An HTML report of the full WebGestalt analysis is available for download. In addition, The GAGE R package [10] is utilized to perform a Gene Set Analysis (GSA) on the full differentially expressed gene lists produced by BART when a GEO experiment is analyzed. BART uses GAGE to determine which KEGG [9] pathways are overrepresented in the down-regulated and up-regulated gene lists for each comparison and displays the results in a table with q-values and other statistical information.

## Results/discussion

### Comparison of popular microarray analysis tools

Since there are multiple extant online microarray analysis tools, we first compared the features of four other top microarray analysis tools to those of BART. In particular, we focused on the types of inputs accepted, the quality control plots that are generated, whether a batch effect correction was possible, how differential expression testing is performed, and whether functional enrichment was possible. We compared BART to 1) GEO2R, a microarray differential expression tool provided by GEO; 2) GDS Tools, curated comparisons provided by GEO; 3) GEO2Enrichr, a recent online tool which uses characteristic direction for differential expression, and shinyGEO, another recent tool (Table 1). Compared to GEO2R, GEO2Enrichr, shinyGEO, and GDS tools, BART functions on a wider variety of microarray platforms and generates more quality control plots.

**Table 1:** Comparison of BART and leading microarray analysis tools with respect to accepted input, data visualization options, and differential expression/post-processing options

	Accepted Inputs				QC Plots				Batch	Differential Expression Test	Functional Enrich. Analysis
	GEO CEL files	Expression Table	User Table	GEO DataSet	Volcano Plot	PCA Plot	Heatmap	Box Plots/Bar Graphs			
BART	**✓**	**✓**	**✓**	**✓**	**✓**	✓	✓	✓	✓	Limma	KEGG
GEO2R	✗	**✓**	✗	**✓**	✗	✗	✗	✓	✗	Limma	✗
GEO2Enrichr	✗	**✓**	**✓**	**✓**	✗	✓	**✓**	✗	✗	Characteristic direction or t-test	Enrichr
shinyGEO	✗	**✓**	✗	**✓**	✗	✗	✗	✗	✗	t-test, one gene at a time	✗
GDS Tools	✗	✗	✗	**✓**	✗	✗	✓	✓	✗	Two-tailed or one-tailed t-test, Value means difference, Rank Means Difference	FLink

Because BART is the only tool that can analyze either CEL files or expression tables from GEO, BART functions on virtually all microarray series on GEO. In addition, only BART and GEO2Enrichr can accept user supplied microarray expression tables which are not published on GEO. BART is the only tool that offers batch effect correction, which is often essential to microarray analysis. BART shares the functionality to perform hierarchical clustering with GDS tools and GEO2Enrichr, but only BART and GEO2Enrichr provide a PCA plot. BART is the only tool that displays a volcano plot, which is useful for quickly visualizing differential expression results. Both BART and GEO2R use limma for differential expression analysis, which is a widely accepted method for fitting and testing microarray data for differentially expressed genes. BART and GEO2R are also the only tools which supply *p*-values and adjusted p-values for full differentially expressed genes. By default, GEO2Enrichr uses a novel method for differential expression testing called characteristic direction which supplies a score for each gene but does not qualify the results based on statistical significance, meaning the user must decide an arbitrary cutoff for the differentially expressed gene list. The other tools use simple t-tests or other basic statistical tests for differential expression testing, however, GDS does not provide *p*-values in its gene lists and shinyGEO provides differential expression results only one gene at a time upon lookup. BART, GEO2Enrichr, and GDS tools provide functionality to extract significant pathways present in the differentially expressed gene lists using different methods.

### BART reanalysis of a monocytopenia study with batch effect correction

**Fig. 2:**
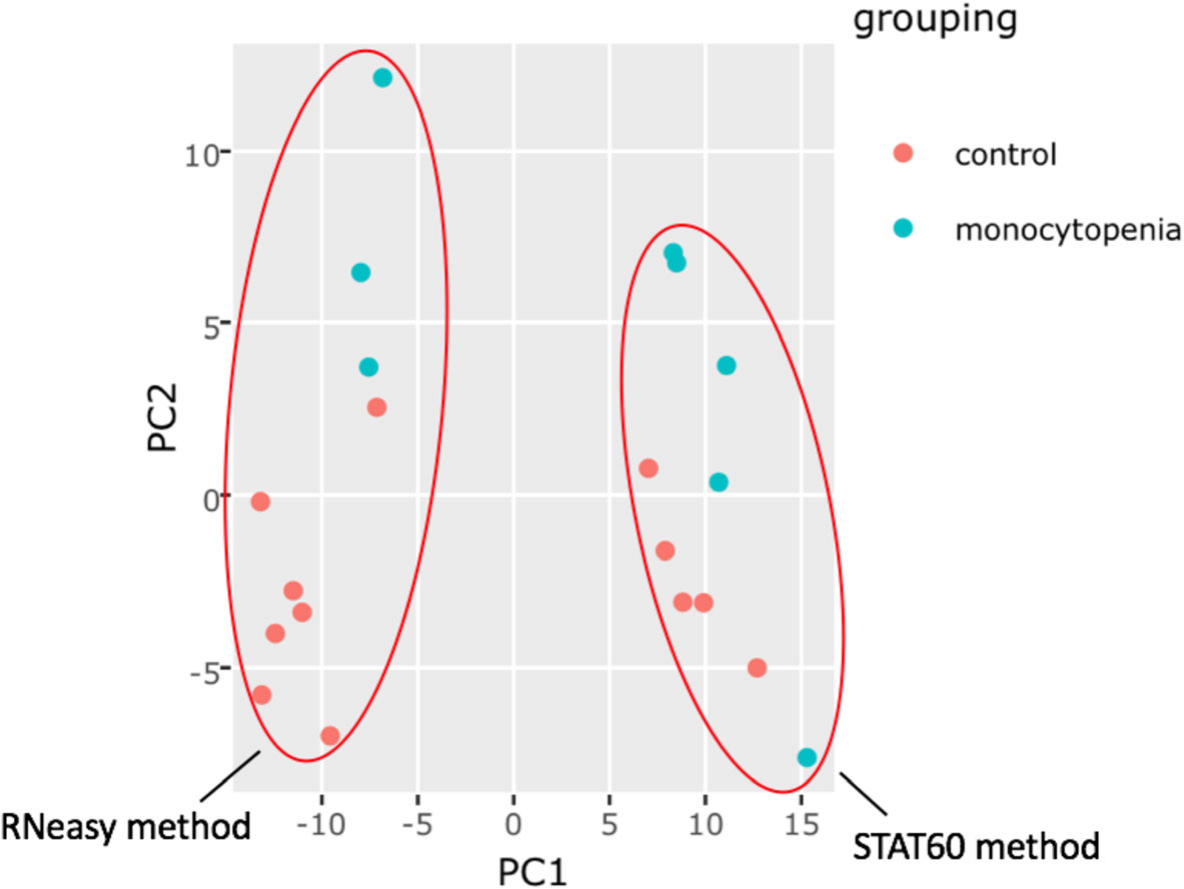
BART PCA plot shows grouping based on RNA isolation method as well as differences due to disease

To demonstrate the utility of BART, we reanalyzed published series GSE16020, a study comparing mycobacterial infection in patients with monocytopenia [15]. First, we used BART to extract phenotypical data from the samples and determine grouping for differential expression testing. We wanted to study the effects of autosomal dominant monocytopenia in polymorphonuclear leukocytes, but noticed that the samples had different RNA isolation methods, which were automatically extracted from the user-supplied annotation by BART. Using BART’s PCA plot feature, we noticed a clear separation between samples with RNA isolated using two different methods (Fig. 2), indicating a considerable batch effect. Because we wanted to determine the effects of monocytopenia only, we utilized BART’s batch effect option to account for the technical variation between the isolation methods. We found that the batch effect correction more than doubled the number of significantly differentially expressed genes BART found from 686 genes to 1,807 genes using an adj. *p*-value < 0.05. Subsequently, BART functional enrichment analysis showed several pathways were upregulated in patients with monocytopenia, including oxidative phosphorylation, lysosomes, and proteasomes. Without batch effect correction, no significantly down or upregulated pathways were found. Because GEO2R does not use raw CEL files and does not have batch effect correction, it found only 212 differentially expressed genes, all of which overlapped with BART’s results. This experiment was not compatible for study with GDS tools or GEO2Enrichr.

### BART reanalysis of a breast cancer study

**Fig. 3:**
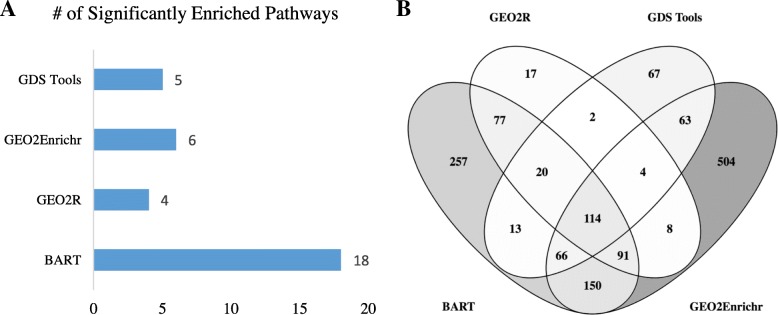
Comparing results of reanalyzing breast cancer datasets with leading microarray analysis tools and BART. **a** Bar graph depicting the number of pathways significantly overrepresented in each differentially expressed gene list using the WebGestAlt KEGG overrepresentation analysis. Pathways were considered significant with an adjusted *p* value less than 0.05. **b** Venn diagram compares the differentially expressed gene lists found with each tool

We next used BART to reanalyze a breast cancer study on GEO (GSE31192). We were interested in comparing normal epithelial cells to tumor epithelial cells and used the raw CEL files available to begin analysis with BART. BART was able to extract 788 differentially expressed genes (adj. *p* value < 0.01) from this comparison. We performed the same analysis with GEO2R, which uses only the user supplied expression table for analysis, and found 333 differentially expressed genes, 302 of which overlapped with BART’s results. We also used GEO2Enrichr to analyze the data, using the maximum cutoff of 1000 genes and the default characteristic direction test for differential expression. Finally, we used GDS tools to analyze this dataset using the default parameter of a two-tailed t-test for differential expression testing, finding 349 differentially expressed genes. To determine whether the results were meaningful, we used WebGestalt [16] to perform pathway analysis using KEGG to determine the number of pathways significantly overrepresented in each differentially expressed gene list (Fig. 3). We found that the differentially expressed gene list from BART resulted in the highest number of enriched pathways (Fig. 3a). In addition, BART’s differentially expressed gene list was the only list enriched for multiple cancer related KEGG pathways, including the PI3K-Akt signaling pathway, pathways in cancer, and the p53 signaling pathway. We also compared the differentially expressed genes for each tool using a Venn diagram [11] (Fig. 3b). Our data shows that the tool used to perform microarray analysis can drastically influence the resulting differentially expressed gene list.

Although BART does not always return different results from GEO2R, we find that BART can outperform GEO2R and other tools using raw CEL files in cases where the expression table uploaded to GEO was not normalized or was normalized using a less appropriate normalization method (Fig. 3). For this dataset, BART has the most overlapping genes with the other tools and the differential expression results seem more meaningful in terms of enriched pathways. BART also provided more quality control plots which were useful for visualizing data.

## Conclusions

A thorough and reliable microarray analysis tool is essential to scientists who are interested in extracting knowledge from the more than 50,000 microarray experiments on GEO, or from their experiments. BART is a free and powerful online microarray analysis tools that allows users without bioinformatics knowledge to analyze microarray data starting from GSE accession ids from GEO, raw CEL files, or expression tables. In addition to flexible input, users can specify custom sample groupings, specify batch ids for downstream correction, generate full lists of all differentially expressed genes between any pairwise comparison using the LIMMA modeling package, and check for enriched pathways among differentially expressed genes. All data tables, heatmaps, PCA plots, and volcano plots are available for download (Fig. 1). We designed BART to be more powerful and flexible than current microarray tools to facilitate meaningful interpretation of array data. BART is uniquely capable of processing raw CEL files and performing RMA normalization instead of relying on preprocessed expression tables. In addition, BART is the only comprehensive tool that offers batch effect correction. BART provides a simple interface and comprehensive analysis for any scientist interested in analyzing microarray data (Table 1). In addition, the flexibility and wealth of features allows users to improve analyses of datasets with batch effects (Fig. 2), or when custom comparisons are required (Fig. 3). BART code is available from https://bitbucket.org/Luisa_amaral/bart and is hosted at bart.salk.edu.

## Availability and requirements

Project name: **BART**

Project home page: **bart.salk.edu**

Operating system(s): **Platform independent**

Programming language: **R**

Other requirements: **None**

License: **MIT license**

Any restrictions to use by non-academics: **No**

## Abbreviations

GDS: GEO DataSet
GEO: Gene expression omnibus
GSE: GEO series
RMA: Robust multichip analysis
